# The Application and Analytical Pathway of Dexmedetomidine in Ischemia/Reperfusion Injury

**DOI:** 10.1155/2019/7158142

**Published:** 2019-12-27

**Authors:** Ying Tang, Changxin Jia, Jianshuai He, Yang Zhao, Huayong Chen, Shilei Wang

**Affiliations:** ^1^Department of Anesthesiology, Affiliated Hospital of Qingdao University, Qingdao, China; ^2^Department of Anesthesiology, Weifang Yidu Central Hospital, Weifang, China; ^3^Department of Anesthesiology, Affiliated Hospital of Qingdao University, Qingdao, China; ^4^Department of Anesthesiology, Weifang Yidu Central Hospital, Weifang, China

## Abstract

Ischemia/reperfusion cerebral injury can cause serious damage to nerve cells. The injured organelles are cleared by autophagy eventually, which is critical for cell survival. Dexmedetomidine is neuroprotective in various ischemia/reperfusion models. Mitochondrial calcium uniporter (MCU) is the most important channel of mitochondrial Ca^2+^ influx into mitochondria, where Ca^2+^ has a potential effect on mitochondrial autophagy. However, the role of MCU in the changes of mitophagy and autophagy caused by dexmedetomidine is unknown. In this study, we constructed an *in vitro* I/R model by subjecting the oxygen and glucose deprivation/reperfusion model to SH-SY5Y cells to mimic the cerebral I/R injury. We found that postconditioning with dexmedetomidine and 3-methyladenine (3MA, an autophagy inhibitor) increased the cell survival meanwhile reduced the production of autophagic vesicles and the expression of LC3 and Beclin 1. This process also increased the expression of BCL-2, P62, and TOM20. After applied with spermine (MCU-specific agonist), the expression of autophagy proteins by dexmedetomidine was reversed, and the same changes were also observed in immunofluorescence. The results of our study suggested that dexmedetomidine can inhibit MCU and reduce excessive mitophagy and autophagy for conferring protection against I/R injury.

## 1. Introduction

Ischemia/reperfusion (I/R) injury is one of the critical pathological processes, which is involved in a series of diseases, such as ischemic shock, cardiac arrest, or cardiac surgery [1, 2]. Due to limited treatments on ischemic diseases, I/R is still a major medical problem that urgently needs to be solved by exploring targeted agents for effective treatments [3]. Recently, many research studies demonstrated that dexmedetomidine (DEX) can exhibit protective effects against I/R injury of several vital organs, including ischemic cerebral injuries [4–7]. The mechanism in the protection of dexmedetomidine may reveal an effective neuroprotective strategy.

Dexmedetomidine is a highly selective agonist of *α*2-adrenergic receptors with clinical sedation and analgesia. Most of mechanism studies involved in the protection of dexmedetomidine were focused on inflammation, Ca^2+^ overload, and signaling pathways. Kim and coauthors showed that dexmedetomidine reduces inflammation by inhibiting nuclear factor, kappa-light-chain-enhancer of activated B cells (NF-*κ*B) [8]. Some studies have reported that dexmedetomidine-induced neuroprotective functions through its activation of *α*2-adrenergic receptors and blocking the voltage-gated calcium channels by inhibiting the Ca^2+^ overload in the cerebral I/R injury [9]. Dexmedetomidine exerts protective effects against ischemia-induced brain injury that may account for the activation of extracellular signal-regulated protein kinase 1 and 2 (ERK1/2) pathways or PI3K-Akt pathways [10, 11]. Recently, dexmedetomidine has been reported to mediate autophagy of cortical neurons to produce neuroprotection after OGD and tMCAO models via upregulation of HIF-1*α* [12]. However, it is still unknown whether dexmedetomidine regulates mitochondrial autophagy or mitophagy in I/R injury.

Mitophagy is a selective pattern of autophagy. Mitophagy plays an important role in mitochondrial quality control and cell survival. Besides its function in quality control, mitophagy has also been proved to be essential to the regulation of mitochondrial turnover, and the adjustment of the amount of organelles to the cellular metabolic requirements [13, 14]. It is also essential for the cells to fight against I/R injury by the timely elimination of dysfunctional mitochondria. However, autophagy appears to be a double-sword in the mechanisms of cellular adaptive system [15]. If the persistent stress induces excessive or prolonged autophagy, the consequential effects may facilitate the necrotic and apoptotic cascades, and thereby result in a cell death [15].

The previous study of our research team has confirmed that inhibition of MCU can inhibit excessive mitophagy by reducing mitochondria fission while maintaining mitochondrial morphology and function, thus protecting the neurocytes from I/R injury [16]. MCU, the most important channel for Ca^2+^, could rapidly accumulate Ca^2+^ across the steep electrochemical gradient [17]. Ca^2+^ signal plays a potential role in modulating and/or triggering mitophagy [18, 19]. It still remains unclear whether reducing excessive mitophagy by inhibiting MCU is associated with the protective effect of dexmedetomidine in I/R injury.

In this study, we hypothesized that dexmedetomidine inhibits excessive mitophagy and autophagy through downregulating MCU in I/R injury model. We constructed an *in vitro* I/R model by subjecting oxygen-glucose deprivation/reperfusion (OGD/R) in SH-SY5Y cells to simulate the process of cerebral I/R injury [20]. We observed that OGD/R triggered the excessive mitophagy and autophagy. These results indicated that dexmedetomidine was neuroprotective by decreasing excessive mitophagy and autophagy. Moreover, dexmedetomidine-induced inhibition of mitophagy and autophagy was found through inhibiting MCU.

## 2. Materials and Methods

All experiments were approved by the institution of ethics committee of Qingdao University Medical College, and all procedures were performed in accordance with the guidelines set by the NIH.

### 2.1. Cell Culture

The techniques that we used were according to the methods described previously by our experimental group [21]. SH-SY5Y cells were purchased from the Central Laboratory, the Affiliated Hospital of Qingdao University. Cells were cultured in DMEM/F12 (Gibco, USA) containing 2 mM L-glutamine, 10% FBS (Gibco, USA), 1% penicillin(100 U/ml of penicillin G), and 1% streptomycin (100 *μ*g/ml of streptomycin). These cells were incubated with 5% CO_2_/95% air and maintained at 37°C in a humidified incubator (Sanyo, Osaka, Japan).

### 2.2. Experimental Groups

The experiment was divided into two major parts. In the first part, cells were divided into 8 groups randomly: (1) control group (C group): cells were cultured normally without any treatment. (2) OGD/R group, in which cells were treated with OGD for 4 h and then returned to normoxic conditions for 18 h. (3) DEX group, in which at the end of OGD, cells were administrated dexmedetomidine (1 *μ*M) (HengRui company, Zhejiang, China. 181217BP) [6, 22] for 18 h reoxygenation. (4) 3MA group, in which at the end of OGD, cells were treated with 3MA (1 mM) for 18 h reoxygenation. (5) DEX + 3MA group: at the end of OGD, cells were administrated dexmedetomidine combined with 3MA for 18 h reoxygenation and concentration as described above, respectively. (6) Rapamycin group (RAPA group): at the end of OGD, cells were treated with rapamycin (10 nM) for 18 h reoxygenation [23]. (7) DEX + RAPA group: at the end of OGD, cells were treated with dexmedetomidine and rapamycin for 18 h reoxygenation and concentration as described above, respectively. (8) DMSO group: at the reoxygenation, the culture medium was mixed with dimethylsulfoxide (0.01%). In the second part, cells were randomly divided into 5 groups: (1) C group. (2) OGD/R group. (3) DEX group. (4) Spermine group (Sper group): cells were treated with spermine (10 *μ*M) for 30 min before OGD and then returned to normoxic conditions. (5) DEX + sper: cells were treated with spermine (10 *μ*M) for 30 min before OGD and treated with DEX (1 *μ*M) at reoxygenation.

### 2.3. OGD/R Model

SH-SY5Y cells were treated with ischemia using an *in vitro* OGD/R model as described previously [21]. To induce OGD, the glucose-free EBSS (Gibco, USA) was used to replace the culture medium. SH-SY5Y cells were cultured in an incubator perfused with 95% N_2_ and 5% CO_2_ at 37°C for 4 h. Then, cells returned to the complete medium by replacing the EBSS for 18 h recovery in normoxic conditions.

### 2.4. Cell Viability Assay

The cells' viability was determined by cell counting kit-8 (CCK-8, QiHaiFuTai biological technology company, China), according to the manufacturer's instructions as described previously [21]. Briefly, 1.5 × 10^4^ cells per well were plated in 96-well plates and cultured at 37^◦^C with 5% CO_2_ in a humidified incubator. 10 *μ*l of CCK-8 solution was added to each well and incubated at 37°C for 2 h. The absorbance was monitored with a microplate reader (Model 550, BIO-RAD, USA) at 450 nm. Culture medium without cells was detected as blank controls. After deducting the absorbance of the blank control, the cells' viability was calculated as dividing the absorbance of experimental groups by that of the control group's. Each experiment was repeated at least 3 times.

### 2.5. Transmission Electron Microscopy (TEM) Observation

TEM examination was performed as described previously [21]. TEM was used to examine the formation of autophagosomes and ultrastructural changes of cell organelles in neurons after OGD/R. Cells were centrifuged at 1000 r/min for 5 min and compacted to cell pallets. After soaking in glutaraldehyde with a 1% (w/v) solution of osmium tetroxide, cells were embedded in Epon812-Araldite. Finally, ultrathin sections were photographed with a JEM-1200EXV transmission electron microscope (JEOL, Tokyo, Japan).

### 2.6. Immunofluorescence

Immunofluorescence was performed as previously described [12]. Briefly, cells were seeded on sterilized glass coverslips, fixed with methanol (4% w: v) for 15 min, permeabilized with Triton-100 (Triton-100, 0.25% in PBS) for 10 min, and blocked with 2% BSA for 1 h. The cells were incubated with rabbit anti-LC3B primary antibodies (1 : 100, Abcam) overnight at 4°C, and then with goat anti-rabbit secondary antibodies (1 : 100, Elabscience) at 37°C for 2 h. Next, cells were stained with DAPI. Finally, images were observed by using a fluorescent microscope (Nikon ECLIPSE80i, Japan).

### 2.7. Western Blot

To detect the protein expression, immunoblotting was carried out as described previously [21]. Protein concentrations were detected via BCA Protein Quantification Kit (Beyotime, China). About 30 *μ*g protein for each sample was loaded and transferred to PVDF membrane. After blocking with 5% nonfat milk for 1 h at room temperature, membranes were treated with mouse mAbs to TOM20 (1 : 1000, Millipore) and rabbit mAbs to Beclin 1 (1 : 2000, Abcam), Bcl-2 (1 : 1000, Abcam), and SQSTM1 (1 : 1000, Abcam) overnight at 4°C. Membranes were washed 3 times with PBST for 15 min each and incubated with respective secondary antibodies for 1 h. The bolts were visualized with an enhanced chemiluminescence kit (VILBER Fusion FX5 Spectra, France). GAPDH (1 : 2000, Abcam) was immunodetected as loading control. Bands were analyzed by ImageJ software.

### 2.8. Statistical Analysis

Each experiment was repeated three times at least. Results were shown as mean ± SD. Differences were evaluated by one-way analysis of variance (ANOVA: three or more groups). When only two groups were compared, an unpaired *t*-test was used. *p* < 0.05 was considered as statistically significance difference. Statistical analyses were performed using SPSS 20.0 Statistics (IBM SPSS Statistics for Version 20.0, IBM Corp, North Castle, NY, USA).

## 3. Results

### 3.1. DEX Increased Viability of Neurons after OGD/R Injury

**Figure 1:**
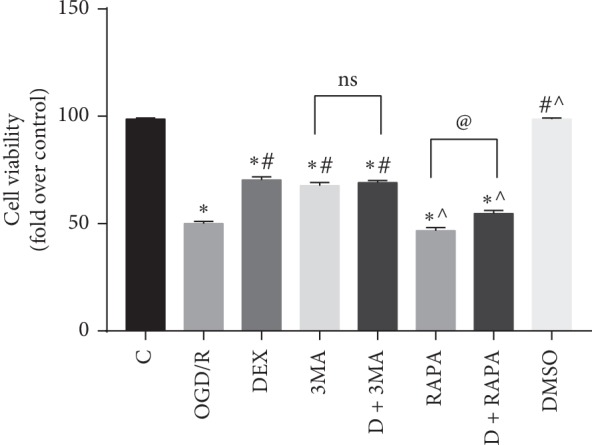
Dexmedetomidine improved viability of neurons. Cell counting kit-8 was used to measure cell viability. ^*∗*^*p* < 0.05, compared with the control group; ^#^*p* < 0.05, compared with the OGD/R group; ^˄^*p* < 0.05, compared with the DEX group; ^@^*p* < 0.05, compared between two indicated groups. Data were recorded as mean ± SD, *n* = 3.

To investigate whether dexmedetomidine-protected cells are against OGD/R injury, CCK-8 assay was used to test the cell viability among different groups (Figure 1). The viability of the OGD/R group, DEX group, 3MA group, DEX + 3MA group, RAPA group, and DEX + RAPA group was significantly lower than that of the control group. The results indicated that DEX, 3MA, and DEX + 3MA groups significantly improved the viability of cells compared with the OGD/R group. Cell viability in the RAPA group and the DEX + RAPA group showed no difference from that of the OGD/R group. The data showed that OGD/R injury caused severe cell injury and DEX could increase the viability of neurons. In addition, this protection was improved/reduced by 3MA/RAPA, respectively.

### 3.2. Dexmedetomidine Inhibited Autophagy and Mitophagy in Neurons after OGD/R Injury

**Figure 2:**
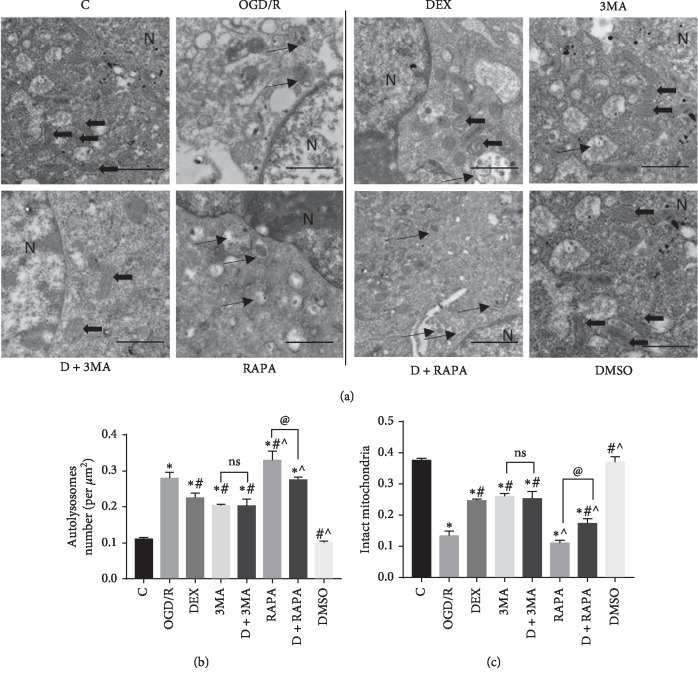
Comparison of ultrastructural changes in different groups. (a) Narrow arrows represent autophagosomes; broad arrows represent intact mitochondria; N represent nucleus. Scale bar = 0.5 *µ*m. The number of autophagosomes (b) and intact mitochondria (c) in each group were counted and analyzed. ^*∗*^*p* < 0.05, compared with the control group; ^#^*p* < 0.05, compared with the OGD/R group; ^˄^*p* < 0.05, compared with the DEX group; ^@^*p* < 0.05, compared between the two indicated groups. Data were recorded as mean ± SD, *n* = 3.

**Figure 3:**
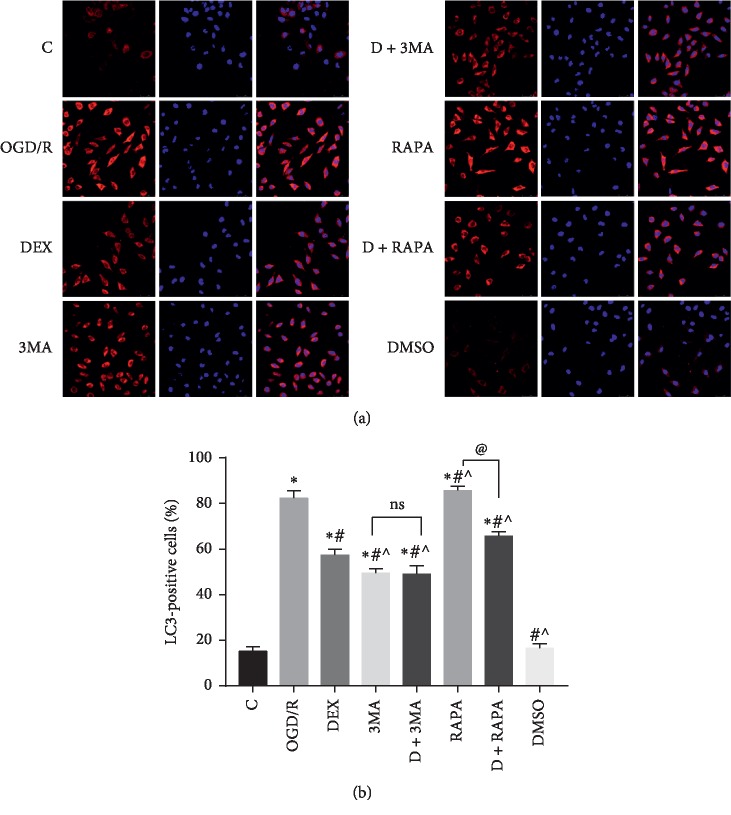
Comparison of the expression of LC3 by immunofluorescence in different groups. (a) Representative LC3 (red) presenting in neuron cells and the blue fluorescence is indicative of cell nucleus. (b) The immunofluorescence intensity of LC3 in neurons was reduced in the DEX group, 3MA group, and DEX + 3MA group, and was increased in the RAPA group compared with the OGD/R group. ^*∗*^*p* < 0.05, compared with the control group; ^#^*p* < 0.05, compared with the OGD/R group; ^˄^*p* < 0.05, compared with the DEX group; ^@^*p* < 0.05, compared between the two indicated groups. Data were recorded as mean ± SD, *n* = 3.

**Figure 4:**
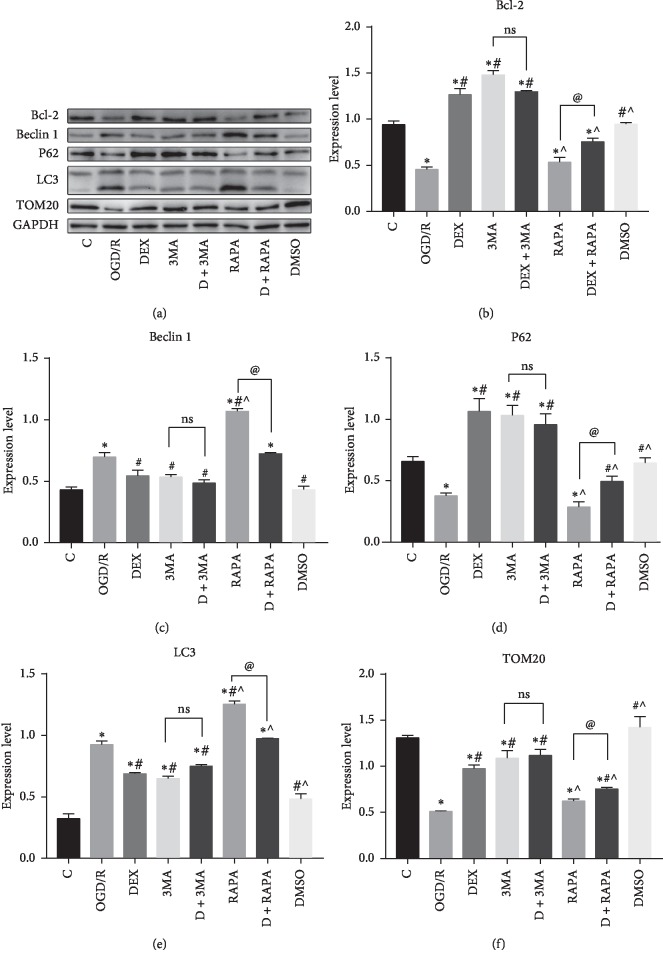
(a) Representative the results of Bcl-2, Beclin 1, P62, LC3, and TOM20 by Western blot. (b–f) The results of Western blot showed that DEX downregulated the activation of autophagy and mitophagy after OGD/R.3MA or RAPA increased or suppressed the autophagic inhibition effect of DEX, respectively. ^*∗*^*p* < 0.05, compared with the control group; ^#^*p* < 0.05, compared with the OGD/R group; ^˄^*p* < 0.05, compared with the DEX group; ^@^*p* < 0.05, compared between the two indicated groups. Data were recorded as mean ± SD, *n* = 3.

A transmission election microscope (TEM) was used to observe the ultrastructure of neurons. It is an important detection for the observation of autophagy and mitophagy [24]. In each group, we detected autophagosomes (narrow arrows, Figure 2(a)) and intact mitochondria (broad arrows, Figure 2(a)). The number of autophagosomes and intact mitochondria in each group was counted and analyzed. These ultrastructural of neurons indicated that autophagy and mitophagy happened and were changed after the OGD/R treatment. Except for the control group, the number of intact mitochondria in other groups was significantly decreased, and autophagosomes were increased evidently. Compared with the OGD/R group, dramatically increased intact mitochondria and decreased autophagosomes were observed in the DEX group, 3MA group, and DEX + 3MA group, and significantly decreased intact mitochondria and increased autophagosomes were observed in the RAPA group. However, dexmedetomidine could partly reverse the effect of RAPA (Figures 2(b) and 2(c)). The above data indicated that dexmedetomidine could inhibit autophagy and mitophagy, but the effect of inhibiting was not strong enough. To gain more solid evidence, we detected the distribution pattern of LC3-positive cells by immunofluorescence (Figure 3(a)). The results clearly showed that the immunofluorescence intensity of LC3 in neurons was reduced in the DEX group, 3MA group, and DEX + 3MA group, compared to the OGD/R group. When the neurons were treated with dexmedetomidine combined with RAPA, the immunofluorescence intensity of LC3 was reduced, compared to the RAPA group (Figure 3(b)). The results showed that autophagic flu was inhibited by DEX. Furthermore, we also detected the expression of markers related to autophagy via Western bolts (Figures 4(a)–4(f)). We noticed that OGD/R robustly increased in LC3 and Beclin 1, and decreased in BCL-2, P62, and TOM20. However, these changes were reversed by the addition of DEX, 3MA, and DEX/3MA combined. Treatment with RAPA made no difference compared with the OGD/R group, which suggested that autophagy and mitophagy were sufficient after OGD/R while dexmedetomidine attenuated these effects. In a word, these data suggested that autophagy and mitophagy were activated after OGD/R injury, and the introduction of dexmedetomidine would reduce excessive autophagy and mitophagy to produce neuroprotection against OGD/R.

### 3.3. DEX Inhibited Autophagy and Mitophagy through Suppressing MCU after OGD/R Injury

**Figure 5:**
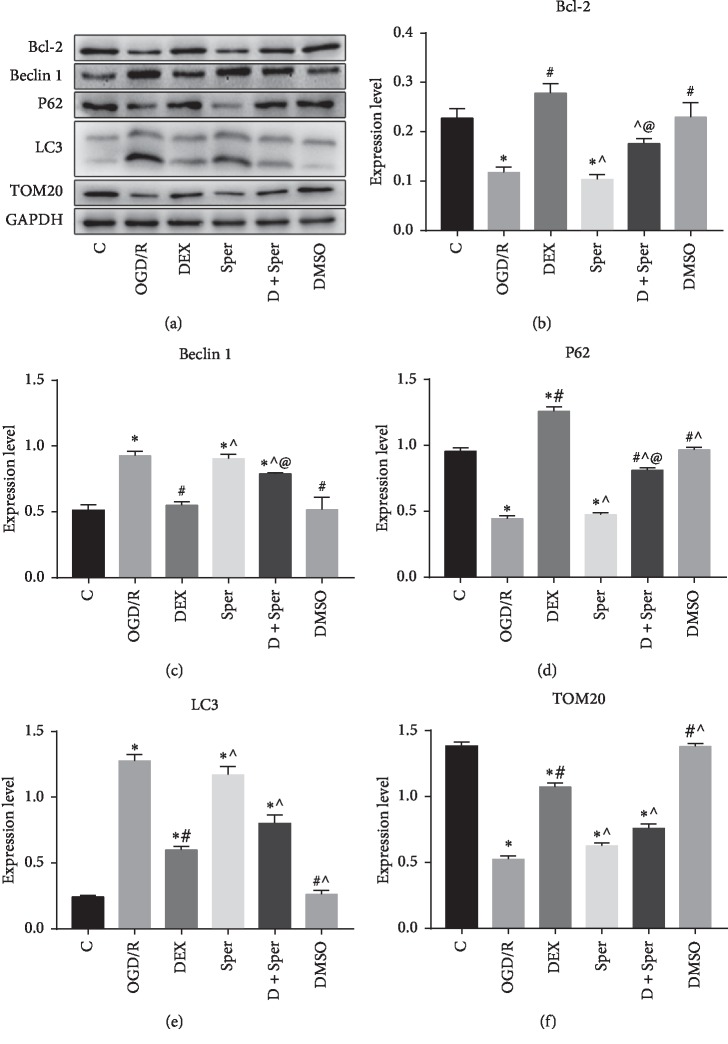
Dexmedetomidine inhibited autophagy and mitophagy through suppressing MCU after OGD/R injury. (a) Comparison of the expression of Bcl-2, beclin 1, P62, LC3, and TOM20 by Western blot. (b–f) The results of Western blot showed that dexmedetomidine downregulated the activation of autophagy and that mitophagy was reversed by spermine after OGD/R. ^*∗*^*p* < 0.05, compared with the control group; ^#^*p* < 0.05, compared with the OGD/R group; ^˄^*p* < 0.05, compared with the DEX group; ^@^*p* < 0.05, compared between the two indicated groups. Data were recorded as mean ± SD, *n* = 3.

Spermine (an agonist of MCU) was applied to investigate the underlying autophagy and mitophagy mechanism of the protective effect of dexmedetomidine after OGD/R injury. The Western blot results demonstrated that treatment with dexmedetomidine obviously increased Bcl-2, TOM20, and p62, and decreased Beclin 1 and LC3 expression. However, the addition of spermine reversed the expression of these proteins (Figures 5(a)-5(f)). Significant differences occurred in the expression of Bcl-2, Beclin 1, and P62 between the group Sper and the group DEX + Sper, respectively, but the expression of TOMA20 and LC3 between the group Sper and the group DEX + Sper was of no significant difference. In a word, these data illustrated that inhibition of MCU might play a crucial role in dexmedetomidine-induced suppression of autophagy and mitophagy.

## 4. Discussion

Since the discovery of autophagy process, it has been reported to be vital in the pathogenesis and progression, as well as the treatment of numerous kinds of diseases [15]. Mitophagy is a selective form of autophagy. Mitophagy is thus considered as the core mechanism of both mitochondrial quality and quantity control [25]. Although there are numerous mechanisms for the protection of dexmedetomidine, the relationship between dexmedetomidine and mitophagy was revealed for the first time. In addition, we also investigated the mechanism of mitophagy mediated by dexmedetomidine. In this study, we presented that dexmedetomidine exerts neuroprotection against I/R injury via inhibition of neuronal autophagy and mitophagy, and this protection could be attenuated by RAPA. We further applied spermine to verify the possible mechanism of dexmedetomidine-induced inhibition of autophagy and mitophagy. When spermine inhibited MCU, dexmedetomidine induced the decrease of mitophagy and autophagy was attenuated. Consequently, these results suggested that the neuroprotective role of dexmedetomidine is mediated by inhibition of autophagy and mitophagy via downregulating MCU after OGD/R injury.

Stroke, also known as a cerebrovascular accident or brain attack, is one of the leading causes of death and disability worldwide [1], but the medical treatment for stroke is very limited [3]. Dexmedetomidine is a commonly used analgesics and sedative in clinic treatment. We choose the postcondition of dexmedetomidine, which is more suitable for most clinical conditions. The previous study of our research group confirmed that dexmedetomidine reduced neuronal apoptosis by inhibiting expressions of Drp1 and Fis1 (mitochondrial fission-related proteins) meanwhile expressions of cytochrome C and caspase 3 [16]. Wang considered that inhibition of mitochondrial fission to reduce the expression of ROS, Drp1, and cytochrome C, and increase the expression of Bcl2 could exhibit neuroprotection against OGD/R injury [26]. Yu also observed this phenomenon in cardiomyocytes [27].

Mitochondria are dynamic organelles that are continuously going through fission and fusion. Fission produces two different forms daughter units. The one with healthier membrane potential will survive, whereas the depolarized daughter mitochondria will be recruited to autophagosomes. Mitochondrial fission may be the upstream condition of mitophagy [23]. Hence, we applied dexmedetomidine with or without 3MA [28] and RAPA, respectively. Treatment with dexmedetomidine combined with or without 3MA exerts neuroprotection. By contrast, once RAPA was applied with dexmedetomidine, the neuroprotection was weakened. The numbers of autophagic vacuoles and partially degraded mitochondria wrapping in double membranes of autophagosomes were characterized by TEM results. These results suggested that mitophagy and autophagy were increased. The changes of autophagic proteins indicated the same results. We analyzed the autophagy-related protein LC3. The rate of LC3-II to LC3-I is usually used to monitor cellular autophagy [29]. An increase in LC3-II protein levels can be applied as a marker of autophagosome formation [30]. Beclin 1 is regarded to promote autophagy [31]. The decrease of Beclin 1 can suppress the autophagic activity in neurons [32]. P62, an autophagic substrate protein, can promote recruitment of damaged mitochondria and depolarized mitochondria to autophagosomes binding with LC3. The decline of P62 represents an increase of autophagic flux [33]. TOM20 is the most important subunit of TOM complex, which is located in the mitochondrial outer membrane. The decline of TOM20 level suggested the decrease of mitochondria quantity [34]. Except for the TEM observation, we also found the increase of TOM20 level compared with OGD/R group, which further indicated that dexmedetomidine reduced the damage of mitochondria. Treatment with RAPA or spermine combined with dexmedetomidine, respectively, neutralized the effect induced by dexmedetomidine. Hence, we suspected that dexmedetomidine inhibited autophagy and mitophagy after OGD/R injury through suppressing MCU. Although the results are consistent with previous conclusions, other researches had different findings from ours [12, 23, 35, 36]. Wu et al. reported that autophagy produced neuroprotection *in vitro* or *in vivo* model suffered from I/R injury via attenuating apoptosis [37]. Liu et al. suggested that the decrease of autophagy flux could enhance the viability of neurons and reduced apoptosis [38]. Dai et al. found that Sirt3 exhibited protection against OGD/R insult by downregulating autophagy through the AMPK-mTOR pathway [39]. Therefore, autophagy and mitophagy may show different effects in cell survival when experiencing different stimulus [22]. Whether autophagy and mitophagy are friends or foes is still controversial [15]. The detailed effect and mechanism of mitophagy and autophagy during I/R injury needs further investigation.

The inner mitochondrial membrane (IMM) is highly selective for calcium. MCU is a vital structure for calcium to pass through IMM [40, 41]. MCU plays an important role in calcium homeostasis. Yu et al. reported that MCU influences cell viability through the changes of autophagy and mitophagy [42]. Mitochondrial Zn^2+^ is an important influencing factor for regulating mitochondrial membrane potential (MMP). Zn^2+^ enters the mitochondrial through the Zip1-MCU complex. Once the mitochondria fail to restore MMP, it will undergo division and mitophagy [43]. Bain believed that accumulation of mitochondrial Zn^2+^ induced PINK/parkin-mediated mitophagy for dysfunctional mitochondria suffering hypoxia-reoxygenation conditions [44]. Therefore, it is possible that dexmedetomidine can inhibit MCU by reducing excessive mitophagy and autophagy for conferring protection against I/R injury.

## 5. Conclusion

Collectively, this study considers that dexmedetomidine can exert neuroprotection through inhibiting mitophagy and autophagy induced by OGD/R. Dexmedetomidine can inhibit MCU by not only reducing excessive mitophagy and autophagy but also preserving mitochondrial morphology and function, thus offering neuroprotective effects.

## Data Availability

The data in this article are true and reliable. The data supporting the results in this article can be found at https://doi.org/10.6084/m9.figshare.11446305.

## References

[1] Haley M. J., Krishnan S., Burrows D. (2017). Acute high-fat feeding leads to disruptions in glucose homeostasis and worsens stroke outcome. *Journal of Cerebral Blood Flow & Metabolism*.

[2] Wei S., Tong J., Xue Q., Liu Y., Xu X. (2017). Effect of puerarin on transcriptome of astrocyte during oxygen-glucose deprivation/reoxygenation injury. *Molecular and Cellular Biochemistry*.

[3] Korin N., Gounis M. J., Wakhloo A. K., Ingber D. E. (2015). Targeted drug delivery to flow-obstructed blood vessels using mechanically activated nanotherapeutics. *JAMA Neurology*.

[4] Breuer T., Bleilevens C., Rossaint R. (2018). Dexmedetomidine impairs diaphragm function and increases oxidative stress but does not aggravate diaphragmatic atrophy in mechanically ventilated rats. *Anesthesiology*.

[5] Wang K., Zhu Y. (2018). Dexmedetomidine protects against oxygen-glucose deprivation/reoxygenation injury-induced apoptosis via the p38 MAPK/ERK signalling pathway. *Journal of International Medical Research*.

[6] Rodríguez-González R., Sobrino T., Veiga S. (2016). Neuroprotective effects of dexmedetomidine conditioning strategies: evidences from an in vitro model of cerebral ischemia. *Life Sciences*.

[7] Shen M., Wang S., Wen X. (2017). Dexmedetomidine exerts neuroprotective effect via the activation of the PI3K/Akt/mTOR signaling pathway in rats with traumatic brain injury. *Biomedicine & Pharmacotherapy*.

[8] Kim E., Kim H.-C., Lee S. (2017). Dexmedetomidine confers neuroprotection against transient global cerebral ischemia/reperfusion injury in rats by inhibiting inflammation through inactivation of the TLR-4/NF-*κ*B pathway. *Neuroscience Letters*.

[9] Cai Y., Xu H., Yan J., Zhang L., Lu Y. (2014). Molecular targets and mechanism of action of dexmedetomidine in treatment of ischemia/reperfusion injury (review). *Molecular Medicine Reports*.

[10] Dahmani S., Paris A., Jannier V. (2008). Dexmedetomidine increases hippocampal phosphorylated extracellular signal-regulated protein kinase 1 and 2 content by an *α*2-Adrenoceptor-independent mechanism. *Anesthesiology*.

[11] Zhang F., Ding T., Yu L., Zhong Y., Dai H., Yan M. (2012). Dexmedetomidine protects against oxygen-glucose deprivation-induced injury through the I2 imidazoline receptor-PI3K/AKT pathway in rat C6 glioma cells. *Journal of Pharmacy and Pharmacology*.

[12] Luo C., Ouyang M. W., Fang Y. Y. (2017). Dexmedetomidine protects mouse brain from ischemia-reperfusion injury via inhibiting neuronal autophagy through up-regulating HIF-1α. *Frontiers in Cellular Neuroscience*.

[13] Youle R. J., Narendra D. P. (2011). Mechanisms of mitophagy. *Nature Reviews Molecular Cell Biology*.

[14] Zhu M., Zhou M., Shi Y., Li W. W. (2012). Effects of echinacoside on MPP+-induced mitochondrial fragmentation, mitophagy and cell apoptosis in SH-SY5Y cells. *Journal of Chinese Integrative Medicine*.

[15] Wang P., Shao B.-Z., Deng Z., Chen S., Yue Z., Miao C.-Y. (2018). Autophagy in ischemic stroke. *Progress in Neurobiology*.

[16] Zhao L., Li S., Wang S., Yu N., Liu J. (2015). The effect of mitochondrial calcium uniporter on mitochondrial fission in hippocampus cells ischemia/reperfusion injury. *Biochemical and Biophysical Research Communications*.

[17] Rizzuto R., De Stefani D., Raffaello A., Mammucari C. (2012). Mitochondria as sensors and regulators of calcium signalling. *Nature Reviews Molecular Cell Biology*.

[18] Rimessi A., Bonora M., Marchi S. (2013). Perturbed mitochondrial Ca^2+^ signals as causes or consequences of mitophagy induction. *Autophagy*.

[19] Calì T., Ottolini D., Negro A., Brini M. (2013). Enhanced parkin levels favor ER-mitochondria crosstalk and guarantee Ca^2+^ transfer to sustain cell bioenergetics. *Biochimica et Biophysica Acta (BBA)-Molecular Basis of Disease*.

[20] Fonseca I., Gordino G., Moreira S. (2017). Tauroursodeoxycholic acid protects against mitochondrial dysfunction and cell death via mitophagy in human neuroblastoma cells. *Molecular Neurobiology*.

[21] Yu S., Zheng S., Leng J., Wang S., Zhao T., Liu J. (2016). Inhibition of mitochondrial calcium uniporter protects neurocytes from ischemia/reperfusion injury via the inhibition of excessive mitophagy. *Neuroscience Letters*.

[22] Dahmani S., Rouelle D., Gressens P., Mantz J. (2010). Characterization of the postconditioning effect of dexmedetomidine in mouse organotypic hippocampal slice cultures exposed to oxygen and glucose deprivation. *Anesthesiology*.

[23] Wang P., Guan Y.-F., Du H., Zhai Q.-W., Su D.-F., Miao C.-Y. (2012). Induction of autophagy contributes to the neuroprotection of nicotinamide phosphoribosyltransferase in cerebral ischemia. *Autophagy*.

[24] Li Q., Zhang T., Wang J. (2014). Rapamycin attenuates mitochondrial dysfunction via activation of mitophagy in experimental ischemic stroke. *Biochemical and Biophysical Research Communications*.

[25] Onphachanh X., Lee H. J., Lim J. R. (2017). Enhancement of high glucose-induced PINK1 expression by melatonin stimulates neuronal cell survival: involvement of MT2/Akt/NF-*κ*B pathway. *Journal of Pineal Research*.

[26] Wang J., Wang P., Li S. (2014). Mdivi-1 prevents apoptosis induced by ischemia-reperfusion injury in primary hippocampal cells via inhibition of reactive oxygen species-activated mitochondrial pathway. *Journal of Stroke and Cerebrovascular Diseases*.

[27] Li Y., Liu X. (2018). Novel insights into the role of mitochondrial fusion and fission in cardiomyocyte apoptosis induced by ischemia/reperfusion. *Journal of Cellular Physiology*.

[28] Luo J. (2014). Autophagy and ethanol neurotoxicity. *Autophagy*.

[29] Knaevelsrud H., Carlsson S. R., Simonsen A. (2013). SNX18 tubulates recycling endosomes for autophagosome biogenesis. *Autophagy*.

[30] Gómez-Sánchez R., Gegg M. E., Bravo-San Pedro J. M. (2014). Mitochondrial impairment increases FL-PINK1 levels by calcium-dependent gene expression. *Neurobiology of Disease*.

[31] Kabeya Y., Mizushima N., Ueno T. (2000). LC3, a mammalian homologue of yeast Apg8p, is localized in autophagosome membranes after processing. *The EMBO Journal*.

[32] Komatsu M., Waguri S., Chiba T. (2006). Loss of autophagy in the central nervous system causes neurodegeneration in mice. *Nature*.

[33] BenYounès A., Tajeddine N., Tailler M. (2011). A fluorescence-microscopic and cytofluorometric system for monitoring the turnover of the autophagic substrate p62/SQSTM1. *Autophagy*.

[34] Zhang X., Yan H., Yuan Y. (2013). Cerebral ischemia-reperfusion-induced autophagy protects against neuronal injury by mitochondrial clearance. *Autophagy*.

[35] Puyal J., Vaslin A., Mottier V., Clarke P. G. H. (2009). Postischemic treatment of neonatal cerebral ischemia should target autophagy. *Annals of Neurology*.

[36] Shi R., Weng J., Zhao L., Li X. M., Gao T. M., Kong J. (2012). Excessive autophagy contributes to neuron death in cerebral ischemia. *CNS Neuroscience & Therapeutics*.

[37] Wu B., Luo H., Zhou X. (2017). Succinate-induced neuronal mitochondrial fission and hexokinase II malfunction in ischemic stroke: therapeutical effects of kaempferol. *Biochimica et Biophysica Acta (BBA) - Molecular Basis of Disease*.

[38] Liu X., Tian F., Wang S., Wang F., Xiong L. (2018). Astrocyte autophagy flux protects neurons against oxygen-glucose deprivation and ischemic/reperfusion injury. *Rejuvenation Research*.

[39] Dai S.-H., Chen T., Li X. (2017). Sirt3 confers protection against neuronal ischemia by inducing autophagy: involvement of the AMPK-mTOR pathway. *Free Radical Biology and Medicine*.

[40] Oxenoid K., Dong Y., Cao C. (2016). Architecture of the mitochondrial calcium uniporter. *Nature*.

[41] Kamer K. J., Mootha V. K. (2015). The molecular era of the mitochondrial calcium uniporter. *Nature Reviews Molecular Cell Biology*.

[42] Yu Z., Chen R., Li M. (2018). Mitochondrial calcium uniporter inhibition provides cardioprotection in pressure overload-induced heart failure through autophagy enhancement. *International Journal of Cardiology*.

[43] Cho H. M., Ryu J. R., Jo Y. (2019). Drp1-Zip1 interaction regulates mitochondrial quality surveillance system. *Molecular Cell*.

[44] Bian X., Teng T., Zhao H. (2018). Zinc prevents mitochondrial superoxide generation by inducing mitophagy in the setting of hypoxia/reoxygenation in cardiac cells. *Free Radical Research*.

